# Supplementary figures and images for: Distinguishing Fine Structure and Summary Representation of Sound Textures from Neural Activity
Source: eNeuro. 2023 Oct 12;10(10):ENEURO.0026-23.2023. doi: 10.1523/ENEURO.0026-23.2023 (PMC10576259; doi:10.1523/ENEURO.0026-23.2023)

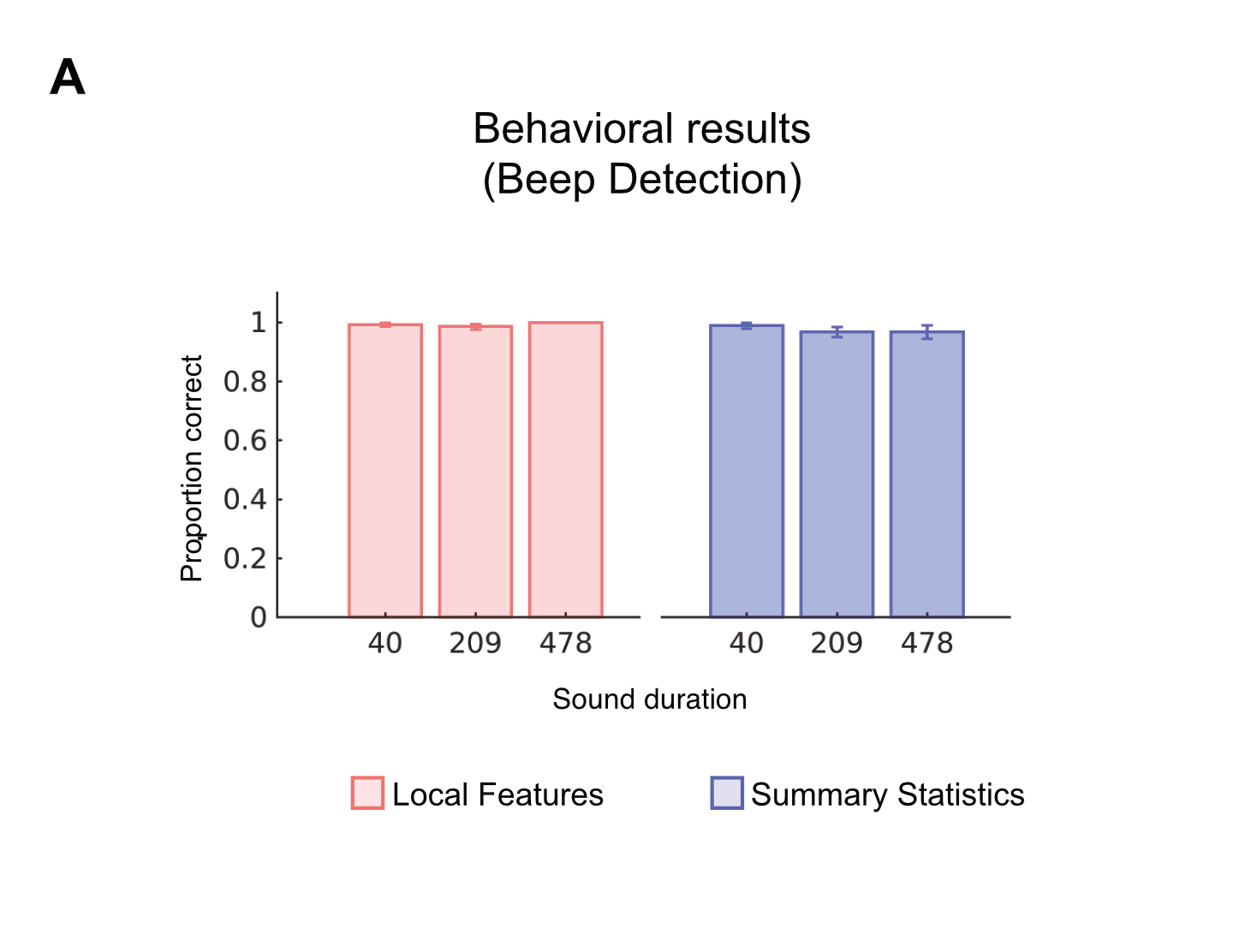

Supplement: Extended Data Figure 1-1 — Behavioral results. Related to Figure 2. A, The group-level average proportion of correct detections of beeps when presented. Bar plots represent average values of hits across all participants. Error bars represent the SEM. No significant difference existed across conditions (all p > 0.05). Download Figure 1-1, TIF file. [file enu-eN-NWR-0026-23-s02.tif]

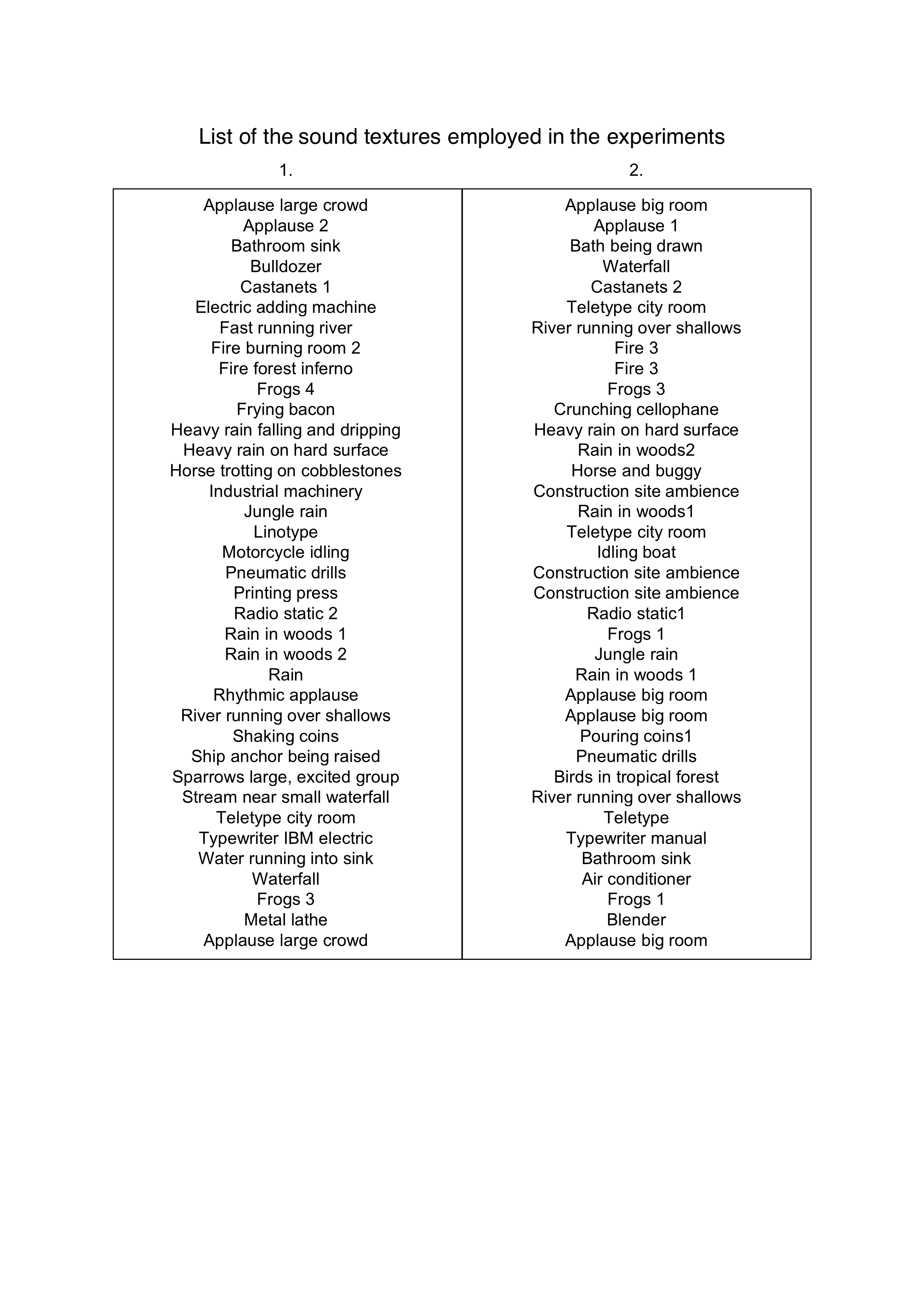

Supplement: Extended Data Figure 1-2 — List of sound textures. Related to Figures 1 and 2. In local features discrimination, for each sound texture in column 1, two synthetic exemplars of the sound texture were selected. One was presented twice (repeated) and the other was presented as the third element of the triplet (novel). In summary statistics discrimination, sound textures were paired according to perceived similarity (McDermott et al., 2013). For each sound texture in column 1, one synthetic exemplar was selected and presented twice. Then, an exemplar of the texture from the corresponding row in column 2 was selected and used as the third element of the triplet (novel). Download Figure 1-2, TIF file. [file enu-eN-NWR-0026-23-s03.tif]

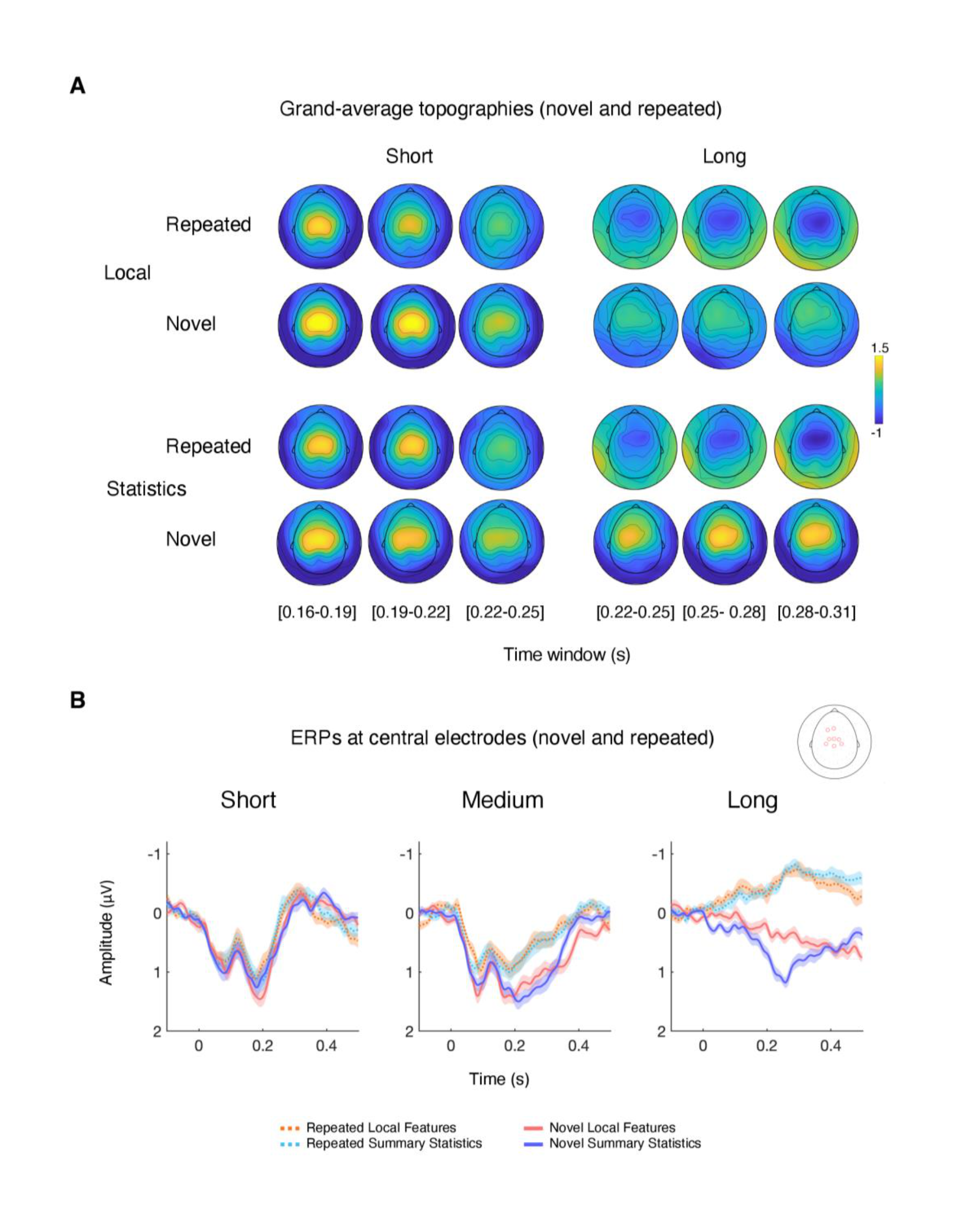

Supplement: Extended Data Figure 2-1 — Auditory evoked response for repeated and novel sounds. Related to Figure 2. A, Grand-average topographies across participants of the responses to standard and oddball sounds for each experiment (local and global discrimination), displayed for short and long durations (478) at latencies of interest. B, Grand-average ERPs across participants of the average amplitude of the central channels displayed in the legend (red circles on the sketch of a scalp). ERPs are shown for both standard and oddball sounds for each experiment and duration. Shaded regions show interpolated SEM at each point. Download Figure 2-1, TIF file. [file enu-eN-NWR-0026-23-s04.tif]
